# Adult Kerion Celsi Caused by *Trichophyton tonsurans* Secondary to Black Dot Tinea Capitis

**DOI:** 10.1007/s11046-022-00701-1

**Published:** 2023-01-08

**Authors:** Qian-ru Ye, Yu-wu Luo, Xin Zhou, Qing-qing Li, Yu-mei Liu

**Affiliations:** Department of Dermatology, Guangzhou Institute of Dermatology, Guangzhou Medical University, No. 56, Hengfu Road, Guangzhou, 510095 China

**Keywords:** Kerion celsi, Trichophyton tonsurans, Tinea capitis, adult

## Abstract

A 34-year-old female patient presented with hair loss due to black dot tinea capitis caused by Trichophyton tonsurans for 6 months. Hair loss progressed to painful swelling for 2 months due to kerion Celsi which may be associated with treatment like topical minoxidil, antibiotic and corticosteroid previously. The patient was treated with oral Itraconazole initially without success but cured by Terbinafine eventually. It’s very interesting that the patient caught kerion celsi secondary to a four-month history of hair loss due to black dot tinea capitis.

A 34-year-old female presented with a 6-month history of hair loss (Fig. 1a) and a 2-month history of painful swelling of the scalp. Her condition had been treated as androgenic alopecia, bacterial folliculitis and seborrheic dermatitis with topical minoxidil, antibiotic and corticosteroid previously for over 2 months, but without improvement. She reported a normal menstrual cycle and a recent haircut at a barbershop prior to hair loss. Additionally, she denied any direct contact with pets and history of tinea of herself and her family members. Physical examination revealed erythematous, boggy, and purulent plaques with abscess formation, greasy scabs and hair loss on her occiput (Fig. 1b). The lesion was tender and the hair pull test was positive. Under dermoscopy, broken hairs, black dots, pustules and scales were observed (Fig. 2a). Direct microscopy of broken hair revealed arthroconidia and hyphae within hair shafts (Fig. 3a). Skin biopsy of scalp lesion revealed arthroconidia within hair shafts (Fig. 3b). Flat, powdery, reddish-brown colonies were observed 2 weeks after incubation on Sabouraud dextrose agar medium (Fig. 4a). Microscopic examination of the smear of colony revealed many inflated, pear-shaped microconidia, borne on matchstick-like stalks (Fig. 4b). This was identified by DNA sequencing of the D1/D2 region of the large subunit of the 28S ribosomal RNA gene as *Trichophyton tonsurans* (GenBank Accession No. ON614136).

**Fig. 1:**
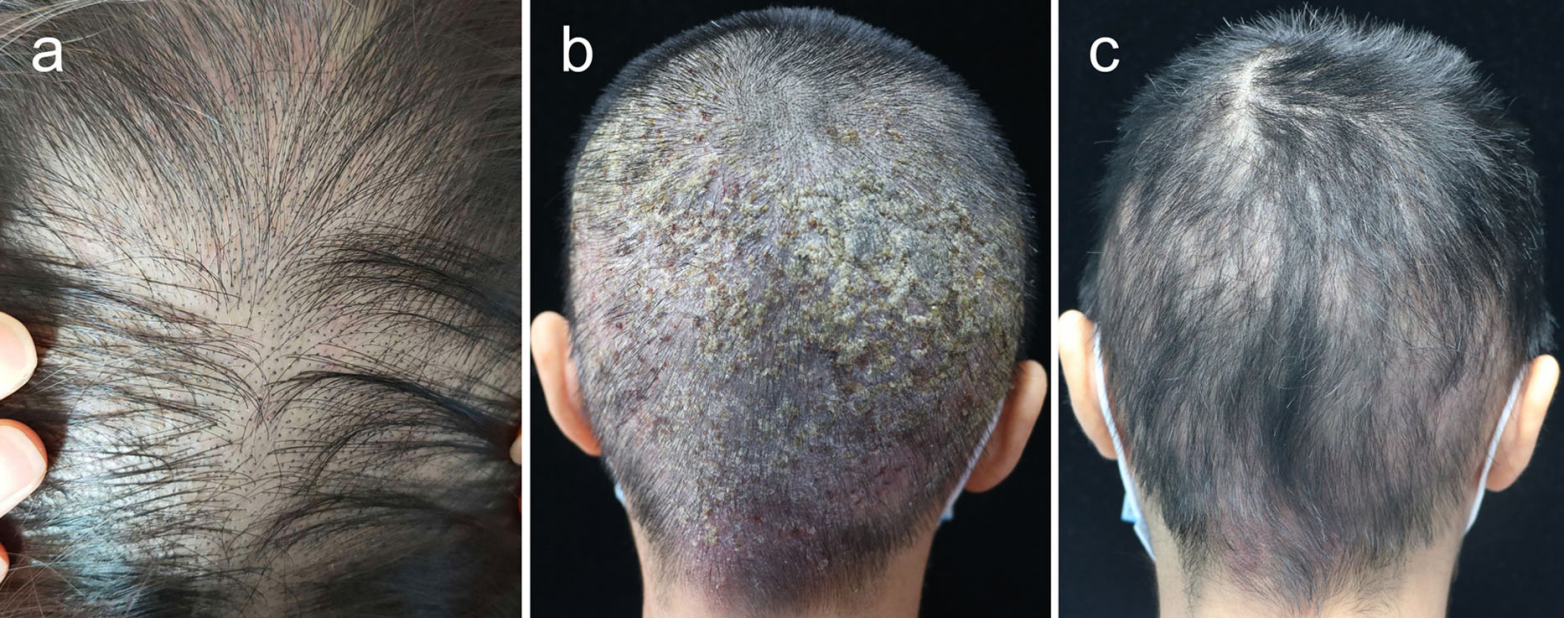
Hair loss at 6 months before visiting our clinic (**a**). Erythematous, boggy, and purulent plaques with abscess formation, greasy scabs on her occiput when visiting us (**b**). Resolution of the abscess and plaques 10 weeks after treatment with Terbinafine (**c**)

**Fig. 2:**
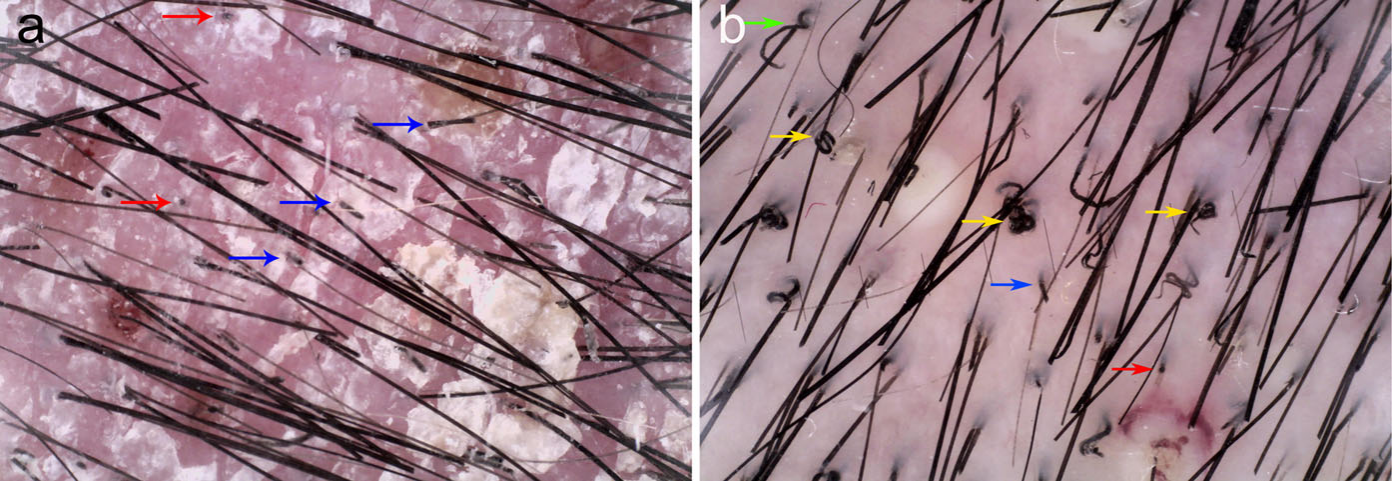
Dermoscopy: broken hairs (blue), black dots (red), comma hairs (green), corkscrew hairs (yellow) and pustules (**a**: Lesions of her occiput when visiting us, **b**: Lesions of her vertex 2 weeks after treatment with Itraconazole)

**Fig. 3:**
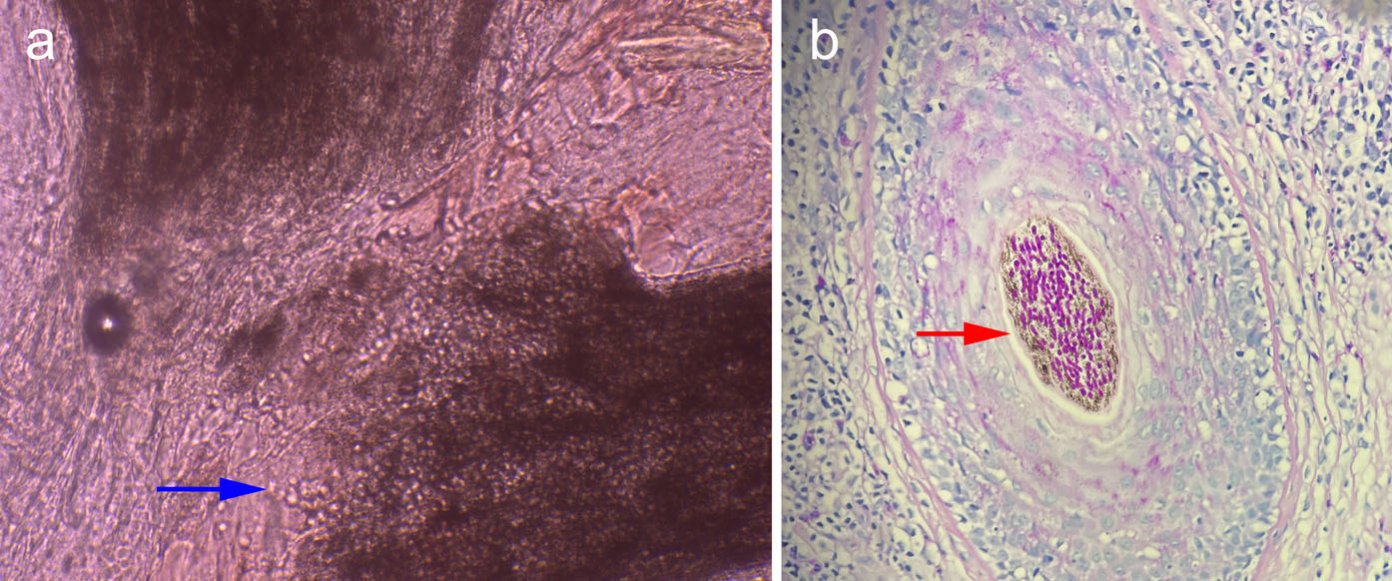
Direct microscopy of broken hair revealed arthroconidia and hyphae (blue) within hair shafts (**a**, 10% Potassium hydroxide smear, × 400). Histopathology: arthroconidia (red) within hair shafts (**b**, Periodic acid-Schiff staining, × 400)

**Fig. 4:**
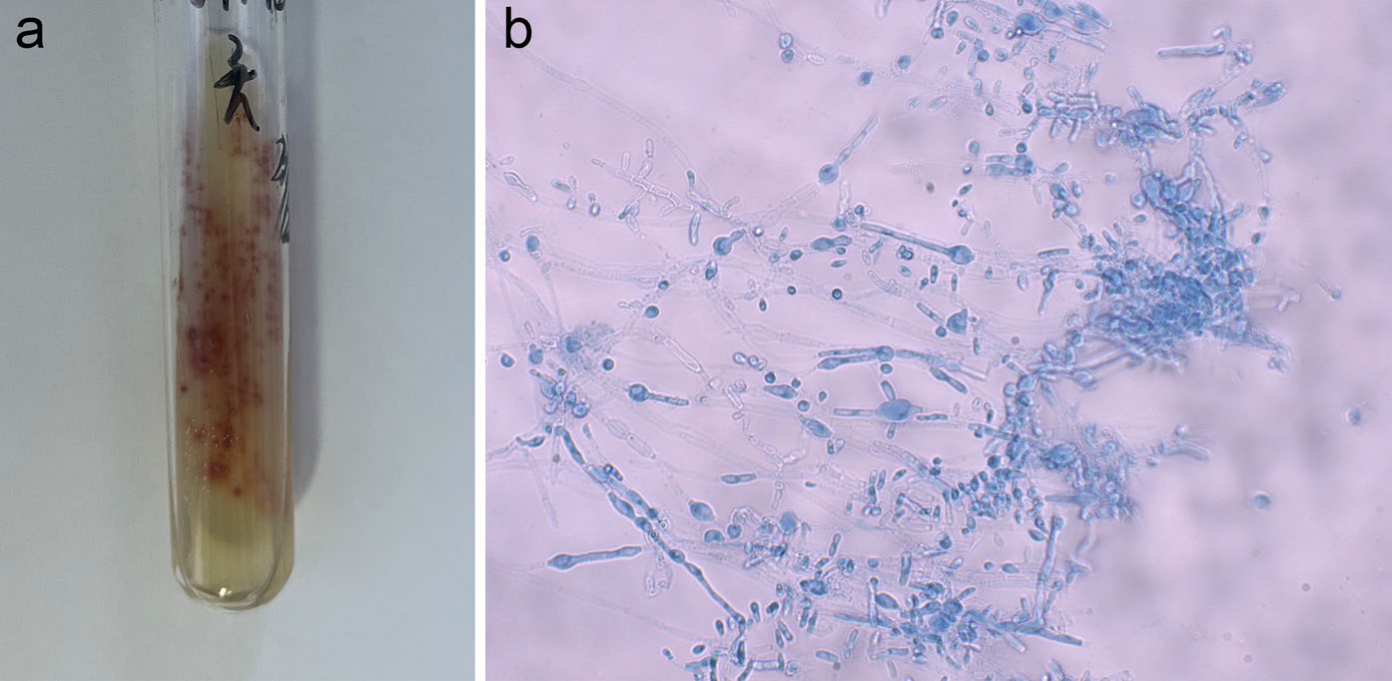
Flat, powdery, reddish brown colonies were observed 2 weeks after incubation on Sabouraud dextrose agar (**a**). Many inflated, pear-shaped microconidia, borne on matchstick-like stalks (**b**, lactophenol cotton blue staining, × 40)

The patient was treated with oral Itraconazole at 200 mg twice a day and prednisolone at 20 mg daily for 2 weeks. Subcutaneous abscesses subsided and the pain was relieved gradually, but her hair loss developed from occiput to vertex. On her vertex, broken hairs, black dots, comma hairs, corkscrew hairs and pustules were seen under dermoscopy at this time (Fig. 2b). Itraconazole was replaced by Terbinafine at 250 mg daily and the lesion resolved after 10 weeks. (Fig. 1c). Subsequent fungal direct microscopy and culturing results were both negative for three times.

Tinea capitis mainly affects preadolescent children. In China, only 7.7% of patients with tinea capitis are adults. Kerion celsi is a severe inflammatory condition of the tinea capitis and is usually caused by zoophilic or geophilic pathogens. Among these organisms, *Microsporum canis* is the most common cause in China. Adult kerion celsi caused by *Trichophyton tonsurans* is exceptionally rare. *Trichophyton tonsurans* is an anthropophilic dermatophyte which usually causes asymptomatic but indolent infections in adults. Our patient may be infected from other people during haircut at a barbershop. Interestingly, the patient was presented with kerion celsi secondary to a 4-month history of black dot tinea capitis. It is possible that her treatment with topical minoxidil, antibiotic and corticosteroid promoted the development from a black dot capitis to kerion celsi. Tinea capitis should be considered in an adult with hair loss, comma hairs and corkscrew hairs under dermoscopy are helpful to differentiate it from androgenic alopecia and seborrheic dermatitis. Current evidence supports that terbinafine is an effective first-line choice for tinea capitis infected with Trichophyton species while itraconazole and fluconazole are alternative but not optimal choices.

